# Evaluation of Functionalized Spider Silk Matrices: Choice of Cell Types and Controls are Important for Detecting Specific Effects

**DOI:** 10.3389/fbioe.2014.00050

**Published:** 2014-11-06

**Authors:** Jan Johansson, Anna Rising

**Affiliations:** ^1^Division for Neurogeriatrics, Department of Neurobiology, Care Sciences and Society (NVS), Center for Alzheimer Research, Karolinska Institutet, Huddinge, Sweden; ^2^Department of Anatomy, Physiology and Biochemistry, Swedish University of Agricultural Sciences, Uppsala, Sweden

**Keywords:** biomaterial, scaffold, matrix, cell-binding motif, RGD, cell culture, compatibility

The ideal scaffold for engineering and regeneration of tissues would be a replica of the extracellular matrix (ECM), which is unique for each tissue type. The scaffold should mimic the mechanical properties of the targeted tissue and serve as matrix for adhesion, growth, migration, and differentiation of endogenous and/or implanted cells. Recent research has highlighted the potential of targeting also the environment of the intermediate states that are formed during tissue repair, since progenitor cells that contribute to tissue formation in a regenerative niche exist in an environment that is different from the final tissue (e.g., the fracture callus that is formed during osteogenesis is softer than mature bone tissue) (Polo-Corrales et al., 2014). In addition, the scaffold should not evoke inappropriate immune responses and should be degradable. To improve cell interactions, ECM-derived cell-binding peptide motifs have been extensively used (Sengupta and Heilshorn, 2010; Maia et al., 2013).

For improving cell attachment to biomaterials, the RGD (Arg-Gly-Asp) peptide motif is commonly used. This peptide is found in fibronectin, vitronectin, bone sialoprotein (BSP), and collagen VI and is recognized by the αvβ3 integrin (Arnaout et al., 2005). The RGD motif exhibits its full binding activity to integrins only when its mobility is restricted in a loop conformation, which can be accomplished *in vitro* by the incorporation of RGD in a cyclic peptide structure (Kumagai et al., 1991; Mohri et al., 1991). In contrast, if the RGD motif is flexible and lacks a stable conformation, it has a much lower affinity to the αvβ3 integrin (Pfaff et al., 1994).

RGD-based peptides have been covalently linked to homopolymers (Brandley and Schnaar, 1988; Kuo and Lauffenburger, 1993; Cook et al., 1997; Bolley et al., 2013), but also covalently incorporated in proteins produced in heterologous hosts, including spider silk-derived proteins (Bini et al., 2006; Wohlrab et al., 2012). Artificial spider silk made from recombinant proteins can form various two- and three-dimensional matrices that hold promise for culture of cells for tissue engineering (Bini et al., 2006; Wohlrab et al., 2012; Wu et al., 2014). These matrices are promising but to realize their full potential, they have to be assembled in a controlled and reproducible way. The recently determined physiological and molecular events that control spider silk formation (Andersson et al., 2014; Kronqvist et al., 2014) have made this realistic for the first time. Functionalization of the silk matrices with cell adhesion motifs via genetic engineering may be a great advantage of the material that likely allows incorporation of cues for adhesion of specific cells, but this has not been fully investigated.

Bini et al. compared films made from a recombinantly produced segment derived from the repetitive part of major ampullate spidroin (MaSp) from *Nephila clavipes* with or without an RGD motif incorporated, and analyzed Ca^2+^ deposition as a measure of cell response and human mesenchymal stem (hMSC) growth (Bini et al., 2006). The authors found that the matrix containing RGD did not show enhanced function as regard cell outcomes and speculated that the lack of RGD effect may be caused by insufficient exposure on the films. Wohlrab et al. studied growth of the cell line BALB/3T3 derived from mouse fibroblasts on films made from a recombinant protein segment from MaSp2 from *Araneus diadematus* (Wohlrab et al., 2012). RGD was either genetically incorporated into the silk protein, or added by linking a cyclic RGD peptide to Cys residues incorporated into the MaSp-derived protein. In both cases, adhesion and proliferation was improved compared to the wild-type protein as well as to a variant in which genetically incorporated RGD was replaced with RGE, a control motif, which binds significantly weaker to integrins. Widhe et al. reported that genetic incorporation of RGD into artificial silk, based on the minispidroin 4RepCT derived from *Euprosthenops australis* MaSp1, improved adherence and growth of human primary fibroblasts compared to 4RepCT films. Incorporation of the control motif RGE was reported to result in decreased attachment and about 50% slower growth rate compared to the RGD-4RepCT (Widhe et al., 2013). However, growth curves for RGD- and RGE-modified matrices were not analyzed.

Wu et al., in contrast, found that the RGD-4RepCT films did not improve attachment of human embryonic stem cells (hESC) compared to wild-type 4RepCT, while incorporation of RGD within the longer vitronectin-derived peptide motif PQVTRGDVFTM resulted in efficient hESC attachment and growth. The effect of the vitronectin-derived peptide motif required integrin binding to the RGD motif, as evidenced by a competition experiment (Wu et al., 2014). The differences between Wohlrab et al. and Widhe et al. on the one hand and Wu et al. on the other hand may depend on that hESC are demanding to culture *in vitro*, while cell lines and fibroblast are robust and will attach to and grow on a wide range of supports and matrices. To test this hypothesis, we performed the same experiments as in Widhe et al. but analyzed the growth curves for human primary fibroblasts on RGD- and RGE-containing films. Surprisingly, we found that human fibroblasts grow equally well on RGD- and RGE-functionalized 4RepCT films (Figure 1).

**Figure 1:**
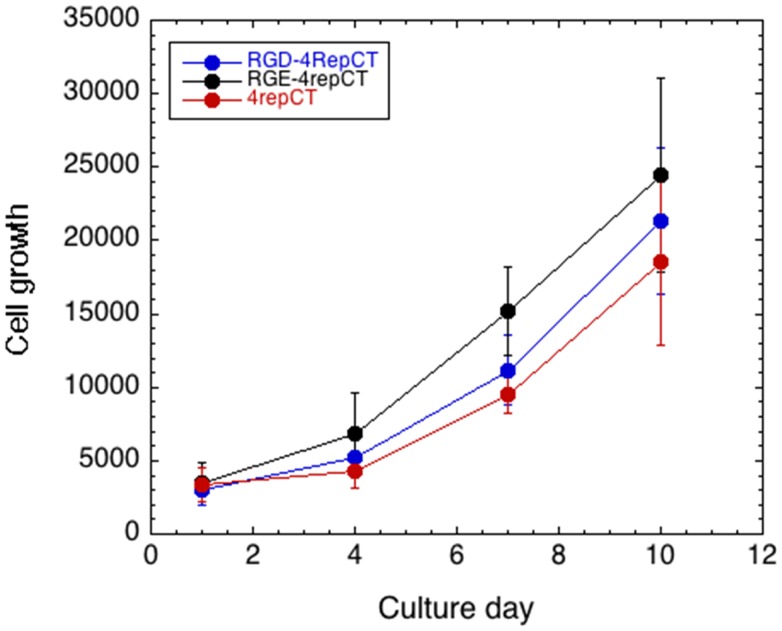
**Cell growth on functionalized spider silk matrices**. Growth curves for primary human fibroblasts grown on RGD-4RepCT, RGE-4RepCT, and 4RepCT films, respectively, as described in Widhe et al. (2013). Cell growth was monitored with Alamar Blue cell viability assay. Each scaffold type was analyzed in hexaplicates, and the results are representative of three independent experiments.

The different results as regard the functional importance of RGD incorporated into spider silk-derived matrices for cell culture described above suggest that care must be taken when evaluating different silk-derived biomaterials. The cells used for culture must certainly be chosen with respect to intended eventual applications of the material, but the robustness of the cells, in particular their sensitivity to growth conditions, must also be considered. Cell types that are more insensitive to culture conditions, like immortalized cell lines or fibroblasts, may be unsuitable for critical testing of novel biomaterials. It is also important to compare matrices that are functionalized with motifs with presumed biological effects, like RGD, with negative controls, as indirect effects of incorporating novel peptide segments, e.g., mediated by altered general properties of the biomaterial, cannot be excluded.

## Conflict of Interest Statement

The authors declare that the research was conducted in the absence of any commercial or financial relationships that could be construed as a potential conflict of interest.

## References

[B1] Andersson M. Chen G. Otikovs M. Landreh M. Nordling K. Kronqvist N. (2014). Carbonic anhydrase generates CO_2_ and H+ that drive spider silk formation via opposite effects on the terminal domains. PLoS Biol. 12:e1001921. 10.1371/journal.pbio.1001921 25093327 PMC4122339

[B2] Arnaout M. A. Mahalingam B. Xiong J. P. (2005). Integrin structure, allostery, and bidirectional signaling. Annu. Rev. Cell Dev. Biol. 21, 381–410. 10.1146/annurev.cellbio.21.090704.151217 16212500

[B3] Bini E. Foo C. W. Huang J. Karageorgiou V. Kitchel B. Kaplan D. L. (2006). RGD-functionalized bioengineered spider dragline silk biomaterial. Biomacromolecules 7, 3139–3145. 10.1021/bm0607877 17096543

[B4] Bolley J. Lalatonne Y. Haddad O. Letourneur D. Soussan M. Perard-Viret J. (2013). Optimized multimodal nanoplatforms for targeting alpha(v)beta3 integrins. Nanoscale 5, 11478–11489. 10.1039/c3nr03763k 24154564

[B5] Brandley B. K. Schnaar R. L. (1988). Covalent attachment of an Arg-Gly-Asp sequence peptide to derivatizable polyacrylamide surfaces: support of fibroblast adhesion and long-term growth. Anal. Biochem. 172, 270–278. 10.1016/0003-2697(88)90442-3 3189771

[B6] Cook A. D. Hrkach J. S. Gao N. N. Johnson I. M. Pajvani U. B. Cannizzaro S. M. (1997). Characterization and development of RGD-peptide-modified poly(lactic acid-co-lysine) as an interactive, resorbable biomaterial. J. Biomed. Mater. Res. 35, 513–523. 10.1002/(SICI)1097-4636(19970615)35:4<513::AID-JBM11>3.0.CO;2-C 9189829

[B7] Kronqvist N. Otikovs M. Chmyrov V. Chen G. Andersson M. Nordling K. (2014). Sequential pH-driven dimerization and stabilization of the N-terminal domain enables rapid spider silk formation. Nat. Commun. 5, 3254. 10.1038/ncomms4254 24510122

[B8] Kumagai H. Tajima M. Ueno Y. Giga-Hama Y. Ohba M. (1991). Effect of cyclic RGD peptide on cell adhesion and tumor metastasis. Biochem. Biophys. Res. Commun. 177, 74–82. 10.1016/0006-291X(91)91950-H 1710455

[B9] Kuo S. C. Lauffenburger D. A. (1993). Relationship between receptor/ligand binding affinity and adhesion strength. Biophys. J. 65, 2191–2200. 10.1016/S0006-3495(93)81277-3 8298043 PMC1225951

[B10] Maia F. R. Bidarra S. J. Granja P. L. Barrias C. C. (2013). Functionalization of biomaterials with small osteoinductive moieties. Acta Biomater. 9, 8773–8789. 10.1016/j.actbio.2013.08.004 23933486

[B11] Mohri H. Hashimoto Y. Ohba M. Kumagai H. Ohkubo T. (1991). Novel effect of cyclicization of the Arg-Gly-Asp-containing peptide on vitronectin binding to platelets. Am. J. Hematol. 37, 14–19. 10.1002/ajh.2830370105 1708943

[B12] Pfaff M. Tangemann K. Muller B. Gurrath M. Muller G. Kessler H. (1994). Selective recognition of cyclic RGD peptides of NMR defined conformation by alpha IIb beta 3, alpha V beta 3, and alpha 5 beta 1 integrins. J. Biol. Chem. 269, 20233–20238. 8051114

[B13] Polo-Corrales L. Latorre-Esteves M. Ramirez-Vick J. E. (2014). Scaffold design for bone regeneration. J. Nanosci. Nanotechnol. 14, 15–56 10.1166/jnn.2014.9127 24730250 PMC3997175

[B14] Sengupta D. Heilshorn S. C. (2010). Protein-engineered biomaterials: highly tunable tissue engineering scaffolds. Tissue Eng. Part B Rev. 16, 285–293. 10.1089/ten.teb.2009.0591 20141386

[B15] Widhe M. Johansson U. Hillerdahl C. O. Hedhammar M. (2013). Recombinant spider silk with cell binding motifs for specific adherence of cells. Biomaterials 34, 8223–8234. 10.1016/j.biomaterials.2013.07.058 23916396

[B16] Wohlrab S. Muller S. Schmidt A. Neubauer S. Kessler H. Leal-Egana A. (2012). Cell adhesion and proliferation on RGD-modified recombinant spider silk proteins. Biomaterials 33, 6650–6659. 10.1016/j.biomaterials.2012.05.069 22727466

[B17] Wu S. Johansson J. Damdimopoulou P. Shahsavani M. Falk A. Hovatta O. (2014). Spider silk for xeno-free long-term self-renewal and differentiation of human pluripotent stem cells. Biomaterials 35, 8496–8502. 10.1016/j.biomaterials.2014.06.039 25043502

